# Role of AMP-Activated Protein Kinase (AMPK) in Female Reproduction: A Review

**DOI:** 10.3390/ijms26146833

**Published:** 2025-07-16

**Authors:** Nurul Ain Kamar Bashah, Adila A. Hamid, Siti Hajar Adam, Farah Hanan Fathihah Jaffar, Izzat Zulhilmi Abd Rahman, Mohd Helmy Mokhtar

**Affiliations:** 1 Department of Physiology, Faculty of Medicine, Universiti Kebangsaan Malaysia, Cheras, Kuala Lumpur 56000, Malaysia; nurulain_0917@yahoo.com (N.A.K.B.); adilahamid@ukm.edu.my (A.A.H.); farahhanan@ukm.edu.my (F.H.F.J.); 2 Preclinical Department, Faculty of Medicine & Defence Health, Universiti Pertahanan Nasional Malaysia, Kuala Lumpur 57000, Malaysia; siti.hajar@upnm.edu.my

**Keywords:** AMP-activated protein kinase (AMPK), folliculogenesis, pregnancy, pre-eclampsia, polycystic ovarian syndrome

## Abstract

The adenosine monophosphate (AMP)-activated protein kinase (AMPK) signalling pathway regulates cell metabolism, inflammation and the immune response. This signalling pathway is essential for maintaining reproductive homeostasis and influencing steroidogenesis, implantation, and embryonic development. The central sensor, AMPK, regulates cell function in response to metabolic stress. The dysregulation of AMPK signalling has been implicated in several female reproductive disorders, including polycystic ovary syndrome (PCOS), endometriosis, infertility, and reproductive ageing. This review provides an overview of the role of AMPK in reproductive function and disorders, as well as potential therapeutic targets to restore balance in this signalling pathway. It discusses AMPK signalling in folliculogenesis, oocyte maturation, pregnancy maintenance, pre-eclampsia (PE) pathogenesis, PCOS, preterm birth, endometriosis, dysmenorrhoea and other disorders related to female reproduction. A deeper understanding of AMPK signalling in these contexts could provide new insights for the development of therapeutic interventions for reproductive health.

## 1. Introduction

AMP-activated protein kinase (AMPK) is a central regulator of cellular energy homeostasis. It is a heterotrimeric serine/threonine protein kinase consisting of a catalytic α-subunit and two regulatory subunits (β and γ) that exist in multiple isoforms and splice variants [1]. This diversity leads to 12 possible heterotrimeric combinations. AMPK regulates metabolic homeostasis throughout the body by responding to hormones and nutrient signalling. In addition, AMPK is activated in response to energy deprivation or stress, such as low glucose levels or hypoxia. This is regulated by the competitive binding of AMP, adenosine diphosphate (ADP), and adenosine triphosphate (ATP) to three sites on the γ-subunit, which modulates the kinase activity of the α-subunit [2,3]. When AMPK is activated, it promotes catabolic pathways that generate ATP and inhibits anabolic pathways that consume ATP, thus restoring cellular energy balance [4,5,6].

Structural evidence shows that the differential binding of nucleotides modulates the activation or inhibition of AMPK and improves our understanding of the mechanisms involved in energy production [3,7]. The binding of AMP and ADP to the γ-regulatory domain modulates the phosphorylation of the AMPK activation loop, allosteric activation (AMP only), and protection from dephosphorylation. In addition, AMPK can regulate metabolism depending on the ratio of AMP and ATP or ADP and ATP [8]. Regulation via adenine nucleotide binding and phosphorylation and interactions with upstream kinases/phosphatases and direct activators lead to conformational changes. Advances in the structural biology of AMPK show that synthetic activators selectively activate certain AMPK isoforms and protect them from dephosphorylation [8].

It has been shown that the activation of AMPK and its related kinases is primarily mediated by liver kinase B1 (LKB1), which is an upstream kinase [9]. These kinases, including NUAK family kinase 1/2 (NUAK1/2), salt-inducible kinase 1/2/3 (SIK1/2/3), maternal embryonic leucine zipper kinase (MELK), and BR serine/threonine kinase 1/2 (BRSK1/2), play an important role in the maintenance of cellular energy homeostasis and the regulation of essential reproductive processes such as embryogenesis, follicle development, and oocyte maturation. In particular, the dysregulation of this signalling axis has been linked to aberrant cellular processes in cancer, including impaired apoptosis and enhanced epithelial–mesenchymal transitions, both of which contribute to tumour progression [10]. 

AMPK is involved in the regulation of reproductive function via several mechanisms and influences hormone production, mitochondrial biogenesis, and cellular proliferation in gonadal tissue [11]. For example, AMPK activation has been shown to regulate steroidogenesis in ovarian granulosa cells and Leydig cells, thereby modulating oestrogen and testosterone production [12]. Moreover, AMPK regulates oocyte maturation and follicle development, which are important for female fertility. The dysregulation of AMPK signalling is increasingly recognised in reproductive disorders. Understanding the role of AMPK signalling pathways in the reproductive system holds great promise for understanding the pathophysiology of reproductive disorders and exploring novel therapeutic strategies. This review, therefore, examines the role of this signalling pathway in reproductive biology and provides insights into potential avenues for future research and intervention.

## 2. AMPK in the Female Reproductive System

As a key enzyme of energy metabolism, AMPK responds to fluctuations in intracellular energy levels by modulating various physiological processes, including glucose uptake, lipid metabolism and mitochondrial function. The properties of AMPK as an energy sensor enable it to couple with the energy status of reproductive function [13]. In recent years, there has been increasing evidence that AMPK plays an important role in the female reproductive system, where it influences ovarian function, uterine receptivity, and overall reproductive longevity [14]. AMPK is widely distributed in reproductive tissues, including the ovaries and uterus, where it regulates reproductive processes such as folliculogenesis, ovulation, implantation, and decidualisation [15]. By responding to metabolic stress and energy availability, AMPK integrates systemic metabolic cues with reproductive function to ensure optimal conditions for fertility. The dysregulation of AMPK signalling has been implicated in reproductive disorders such as PCOS, premature ovarian failure, and endometrial dysfunctions, highlighting its importance in female reproductive health. Understanding the role of AMPK in reproductive physiology may provide valuable insights into the potential therapeutic targets for reproductive disorders.

### 2.1. AMPK in Female Reproductive Function

#### 2.1.1. AMPK and Hypothalamus Pituitary Gonadal Axis

The gonadotropin-releasing hormone (GnRH) stimulates the release of luteinising hormone (LH) from the pituitary gland, which is essential for reproductive function. Ependymocytes are cells that sense energy and regulate reproduction in animals by responding to changes in extracellular glucose levels and expressing pancreatic-type glucokinase and glucose transporter 2 [16]. When AMPK is activated in the ependymocytes of the lower brainstem in response to low energy levels, it suppresses the release of GnRH and LH, thus impairing reproductive processes [12,16]. In addition, a previous study has shown that the administration of an AMPK activator, 5-Aminoimidazole-4-carboxamide ribonucleotide (AICAR) resulted in reduced pulsatile LH release in female rats [16]. This suggests that AMPK can influence the timing of female reproduction and that GnRH neurons play an important role in regulating LH secretion [17]. Furthermore, the inhibition of the mechanistic target of rapamycin (mTOR) signalling pathway by AMPK in low-energy states leads to the reduced production of reproductive hormones, highlighting its role in the adaptation of reproductive functions to energy availability [18]. Therefore, understanding energy-sensitive signalling pathways could provide valuable insights into female reproductive health, particularly in obesity and metabolic syndrome, which impact fertility.

AMPK in GnRH neurons mediates the metabolic suppression of LH surges by hindbrain lactoprivine signalling, but lactate restores GnRH expression without affecting AMPK activity in steroid-primed ovariectomised female rats exposed to insulin-induced hypoglycaemia [19]. In this study, hypoglycaemia inhibited the LH surge in steroid-primed female rats, but this inhibition was reversed by lactate infusion into the hindbrain. Hypoglycaemia decreased GnRH-I protein and phosphorylated-AMPK (Pampk) levels in rostral preoptic GnRH neurons. In addition, lactate restored GnRH-I protein levels but had no effect on hypoglycaemia-induced GnRH AMPK activation.

In addition to its role in metabolism, AMPK exerts notable anti-inflammatory effects and contributes to the maintenance of a stable and physiologically balanced microenvironment in the gonads. This is particularly significant, as chronic or excessive inflammation is known to impair fertility and disrupt normal reproductive processes. By attenuating inflammatory responses, AMPK helps maintain optimal gonadal activity and supports overall reproductive health. Furthermore, AMPK is involved in modulating the interaction between germ cells and their supporting somatic cells, which are fundamental for successful reproduction. In particular, AMPK influences germ cell morphology and nuclear maturation, which explains its importance in the formation and development of functional germ cells [12,20].

Inflammation can disrupt the hormonal balance, which is important for reproductive function. AMPK plays a regulatory role in maintaining this balance, which is crucial for processes such as ovulation. By modulating inflammatory responses, AMPK supports the hormonal signalling necessary for fertility [12]. AMPK also plays a crucial role in the regulation of progesterone production in the corpus luteum of cattle, the activation of which reduces progesterone secretion [21,22]. Furthermore, the activation of AMPK appears to be a key factor in the response of the mature corpus luteum to prostaglandin F2α (PGF2α), a process that is essential for the involution of the corpus luteum [21]. Thus, targeting AMPK could be a potential strategy to improve reproductive management, particularly to improve oestrus synchronisation. In addition, AMPK is an important mediator of the effects of PGF2α on progesterone production [23]. In vitro studies have shown that AMPK activators, including metformin and AICAR, significantly reduce progesterone synthesis in the mature corpus luteum, suggesting that AMPK activation inhibits progesterone production, which is a crucial aspect of luteal function and involution [23]. In addition, there may be therapeutic applications for the treatment of luteal insufficiency in women undergoing in vitro fertilisation.

#### 2.1.2. AMPK in Folliculogenesis and Oocyte Maturation

AMPK also plays a crucial role in female gametogenesis. Folliculogenesis takes place in a highly dynamic and regulated ovarian microenvironment. In mammals, the ovary consists of follicles, which are its basic functional units. Follicular development begins in humans during the foetal period and in rodents during the neonatal period with the formation of primordial follicles. After activation, primordial follicles are characterised by a single layer of flattened granulosa cells that envelop the oocyte. The follicles pass through various stages of development and develop into primary, secondary and, finally, antral follicles [24].

In women, the preovulatory or Graafian follicles are the main source of cyclical oestrogen secretion from the ovaries. Triggered by the preovulatory gonadotrophin surge in each reproductive cycle, the dominant Graafian follicle releases a mature oocyte for potential fertilisation. The remaining theca and granulosa cells differentiate into the corpus luteum, which then produces circulating progesterone [24]. The cyclic recruitment of follicles, neoangiogenesis, spatial reorganisation, follicular atresia, and ovulation are controlled by a complex interplay between mechanical forces and molecular signalling pathways [25]. These processes involve both structural and functional changes in the developing follicle and its surrounding ovarian tissue, which are essential for the generation of oocytes that support pre-implantation development up to the blastocyst stage [26].

Studies have shown that AMPK activity influences follicular development, including granulosa cell proliferation and oocyte meiotic progression. Oocyte quality and maturation are not only critical for successful fertilisation and embryonic development but also influence long-term foetal growth and developmental outcomes [27,28]. As female fertility declines with age, this reflects a decline in the quality and quantity of oocytes [28,29]. The activation of AMPK in the ovary has been linked to the modulation of follicular growth under metabolic stress and may mediate the effects of metabolic disorders associated with female infertility, such as obesity and PCOS.

The study by Lu et al. (2017) in mice showed that AMPK inhibits mTOR signalling activities via the AMPK/mTOR pathway [30]. AMPK inhibition stimulates follicle development in mouse ovaries via the AMPK-mTOR/Hippo-Yes-associated protein (AMPK-mTOR/Hippo-YAP) interaction. AMPK inhibition increases ovulation and oocyte fertilisation during in situ intrabursal injection with gonadotropin stimulation in mouse ovaries. In addition, AMPK has both positive and negative effects on angiogenesis [31]. The differential effects of AMPK on angiogenesis are mediated by cell-specific responses, environmental factors, and the balance between pro- and anti-angiogenic signalling downstream of AMPK [32]. AMPK activation regulates angiogenesis by promoting stress-induced autophagy, stabilising hypoxia-inducible factor-1 alpha (HIF-1α) and increasing vascular endothelial growth factor (VEGF) expression. At the same time, AMPK can inhibit the angiogenesis caused by tumour-promoting and pro-metastatic factors such as phosphoinositide 3-kinase/protein kinase B/mTOR (PI3K/Akt/mTOR), hepatocyte growth factor (HGF), and transforming growth factor-beta/bone morphogenetic protein (TGF-β/BMP) signalling pathways. It has been shown that AMPK inhibition promotes ovarian angiogenesis via the AMPK–HIF-1α/vascular endothelial growth factor A/vascular endothelial growth factor receptor 2/connective tissue growth factor (AMPK-Hif-1α/Vegfa/Vegfr2/Ctgf) signalling pathway [30,33]. Furthermore, treatment with compound C, an AMPK inhibitor, increased the number of ovulated oocytes that were successfully fertilised and resulted in healthy offspring. This shows that AMPK inhibition promotes folliculogenesis and angiogenesis in the ovaries and thus supports ovarian development.

In addition, AMPK regulates ovarian primordial follicle activation via the AMPK- Wingless-related integration site–forkhead box O-β-catenin) (AMPK–Wnt–FOXO–β-catenin) signalling pathway in murine ovaries [34]. The pharmacological modulation of metformin and dorsomorphin in vitro showed that AMPK activation using metformin inhibited primordial follicle activation in the ovarian cortex of mice and humans, whereas AMPK inhibition using dorsomorphin promoted activation. AMPK inhibition reduces β-catenin phosphorylation, suggesting that dorsomorphin reduces β-catenin phosphorylation and promotes follicle activation through the AMPK–β-catenin interaction [35]. In addition, AMPK inhibition regulates Wnt and FOXO gene expression in mouse ovaries via the AMPK-Wnt-FOXO pathway [36]. This increased Wnt and FOXO gene expression after AMPK inhibition supports follicular activation. However, AMPK inhibition does not induce oxidative stress or apoptosis in mouse ovaries via the AMPK–reactive oxygen species (AMPK-ROS) mitochondrial function. Ovaries treated with dorsomorphin showed no increase in ROS, apoptosis, or mitochondrial dysfunction in mouse ovaries [37,38,39].

In a recent study it was shown that AMPK plays a role in the regulation of oxidative stress, mitochondrial function, and follicle development in preantral follicles through AMPK/peroxisome proliferator-activated receptor gamma coactivator-1 alpha (AMPK/PGC-1α), nuclear factor erythroid 2–related factor 2/heme oxygenase-1 (NRF2/HO-1), mitophagy (PTEN-induced kinase 1; (PINK1), Parkin, and microtubule-associated protein 1 light chain 3-II; LC3-II), mitochondrial biogenesis (nuclear respiratory factor 1/transcription factor A mitochondrial; NRF1/TFAM), apoptosis (B-cell lymphoma 2; Bcl-2, Bcl-2-associated X protein; Bax, tumor protein P53; P53, cleaved caspase 3), and steroidogenesis (steroidogenic acute regulatory protein; StAR and cytochrome P450 side-chain cleavage enzyme; P450scc) [40,41]. In this study, astaxanthin was shown to promote antrum formation, oocyte maturation, follicle attachment, and estradiol secretion. This study also showed an increased expression of proteins involved in the AMPK signalling pathway, markers of mitochondrial biogenesis and antioxidant defence proteins. However, these effects were reversed by an AMPK inhibitor, confirming the involvement of the AMPK signalling pathway.

In maturing bovine oocytes, AMPK and β-fatty acid oxidation enzymes (carnitine palmitoyltransferase 1A; CPT1A, carnitine palmitoyltransferase 1B; CPT1B and carnitine palmitoyltransferase 2; CPT2) play a key role in the regulation of lipid metabolism and mitochondrial function [42]. The study showed that β-aminoisobutyric acid (BAIBA) improved blastocyst formation and embryo quality without affecting the extrusion rate of the first polar body. In addition, BAIBA regulated the expression of CPT1A, CPT1B, and CPT2, which promoted fatty acid β-oxidation, reduced lipid accumulation and improved mitochondrial membrane potential. Importantly, increased AMPK phosphorylation was observed, and the pharmacological inhibition of AMPK blocked BAIBA-induced improvements in lipid metabolism, resulting in a decreased oocyte maturation rate, zygote cleavage, and blastocyst formation [42]. It was, therefore, hypothesised that BAIBA improves the developmental competence of oocytes through the AMPK-mediated regulation of lipid metabolism.

Another recent study by Cheng et al. (2025) on the maternal exposure of pregnant Kunming mice to polystyrene nanoplastics during gestation and lactation showed that AMPK plays a role in regulating primary follicle activation and ovarian function in response to exposure to nanoplastics through AKT-FOXO3a, AMPK, mTOR, and calcium/calmodulin-dependent protein kinase II beta (CAMKIIβ) [43]. It has been shown that the downregulation of AMPK phosphorylation and upregulation of mTOR activity are associated with the activation of the AKT-FOXO3a signalling pathway.

AMPK also supports the survival and proliferation of gonadal cells, including granulosa cells in women, which are important for the development of oocytes. By dampening inflammation, AMPK promotes a more favourable microenvironment for these cells, which is crucial for successful reproduction [12]. In addition, AMPK plays a key role in regulating mitochondrial function, which is essential for cellular energy production. Mitochondrial health is particularly important for cell survival and function in the gonads. By promoting mitochondrial integrity and reducing inflammation, AMPK ensures that gonadal cells produce the energy they need to effectively fulfil their physiological functions [1].

#### 2.1.3. AMPK in Pregnancy

AMPK plays a critical role in maternal and foetal wellbeing by influencing cell growth, nutrient transport, angiogenesis, and the response to oxidative stress for a healthy pregnancy. Impaired AMPK signalling may contribute to the metabolic and vascular abnormalities observed in pregnancy, including mitochondrial dysfunction, increased oxidative stress, and dysregulated trophoblast invasion [44,45,46].

AMPK expression is found throughout the female reproductive system and is important for the maintenance of female fertility. There is evidence that the removal of AMPK subunits in experimental mice leads to smaller litters and an early decline in reproductive capacity [14]. AMPK is also essential for the postpartum endometrium, as its absence leads to severe fibrosis and an impaired uterine structure [47]. In addition, AMPK activity is essential for endometrial remodelling, which is required for successful implantation and pregnancy. AMPK also regulates the response to steroid hormones, the proliferation of epithelial cells and the receptivity of the uterus during implantation [48]. The dysregulation of AMPK can impair uterine receptivity, trigger uterine inflammation, and alter the timing of embryo implantation [15,49].

In a mouse model, AMPK was found to regulate embryo implantation and survival under environmental stress induced by harmful environmental exposure to carbon disulphide via the AKT/AMPK/mTOR signalling pathway [50]. Supplementation with N-carbamoyl glutamic acid reversed these effects by increasing mTOR and phosphorylated protein kinase B (pAKT) levels and decreasing pAMPK levels; it also significantly increased the implantation rate of embryos. This suggests that mTOR activity is regulated by AMPK, the upstream molecule of the mTOR signalling pathway, and it has been demonstrated that AMPK can regulate embryo implantation during the normal fertility process [50,51]. This mechanistic relationship is also supported by clinical findings that showed differential changes in placental concentrations of total and phosphorylated Akt, AMPKα, and mTOR in women with normal pregnancies compared to those with pregnancies complicated by foetal growth restriction (FGR) or gestational diabetes (GDM) with children large-for-gestational-age (LGA) [52]. Functional validation using primary cytotrophoblasts exposed to oxygen–glucose deprivation (OGD) confirmed that Akt and AMPK signalling pathways regulate trophoblast mTOR activity under pathological conditions. Taken together, these in vivo and clinical data highlight the critical role of the AMPK–mTOR signalling pathway in controlling functions and its potential dysregulation in pregnancy complications such as FGR and GDM.

AMPK activation regulates the mTOR-mediated reprogramming of glycolysis, promotes Treg differentiation and improves pregnancy outcomes both in mouse models of unexplained recurrent spontaneous abortion (URSA) and in clinical decidual tissue from URSA patients [53]. The study revealed significant differences in gene expression related to pregnancy-associated signalling pathways, with increased Th17 cell differentiation observed in URSA samples. The activation of AMPK and inhibition of glycolysis significantly reduced the abortion rate and enhanced Treg differentiation while suppressing Th17 differentiation [54]. In addition, treatment with metformin and 2-deoxy-D-glucose (2-DG) further promoted Treg differentiation, restored immune balance, and improved pregnancy outcomes. These findings suggest that the AMPK-mTOR signalling pathway regulates glycolysis-driven immune reprogramming and that Met/2-DG therapies may offer promising clinical interventions for URSA [55].

In addition, AMPK signalling modulates hypothalamic fatty acid metabolism and brown adipose tissue thermogenesis, which contribute to hyperphagia and regulate energy balance during pregnancy in rats [56]. The findings demonstrated that pregnancy alters hypothalamic AMPK signalling, resulting in reduced sensitivity to anorexigenic and thermogenic mechanisms. This adaptation promotes hyperphagia and facilitates energy storage to support foetal development.

#### 2.1.4. AMPK in Ovarian Ageing

Ovarian ageing is a progressive and continuous physiological process characterised by a decrease in both the quantity and quality of ovarian follicles, eventually leading to decreased ovarian function and menopause. Ovarian reserves play a central role throughout a woman’s life, with reduced ovarian reserves being closely linked to reduced fertility. It influences reproductive capacity in earlier years and overall health in later stages [57].

AMPK has been shown to regulate mitophagy and glycophagy in D-galactose (D-Gal)-induced senescent granulosa cells (GCs) in ageing chickens. In this study, follicle-stimulating hormone (FSH) was shown to stimulate mitophagy, reduce mitochondrial oedema, and increase the colocalisation of mitophagosomes with mitochondrial light chain 3 (LC3) through the activation of AMPK-PI3K/AKT pathways. The disruption of FSH-mediated autophagy impaired GC proliferation and glycolysis, while the simultaneous inhibition of PI3K/AKT and AMPK abolished the protective effect on ovarian energy regulation. These results suggest that FSH prevents ovarian ageing by promoting AMPK- and PI3K/AKT-mediated mitophagy and glycophagy [58].

Ageing has been shown to lead to mitochondrial abnormalities in oocytes that impair fertility. A previous study has shown that the transplantation of human amniotic mesenchymal stem cells (hAMSCs) in mice with age-related diminished ovarian reserves (AR-DORs) led to a significant improvement in ovarian function, while the apoptosis of granulosa and stromal cells in the ovaries was significantly reduced. In addition, both the expression of AMPK and the ratio of phosphorylated FoxO3a to total FoxO3a were significantly increased, demonstrating the crucial role of the AMPK/FoxO3a signalling pathway in improving ovarian function [59].

**Figure 1:**
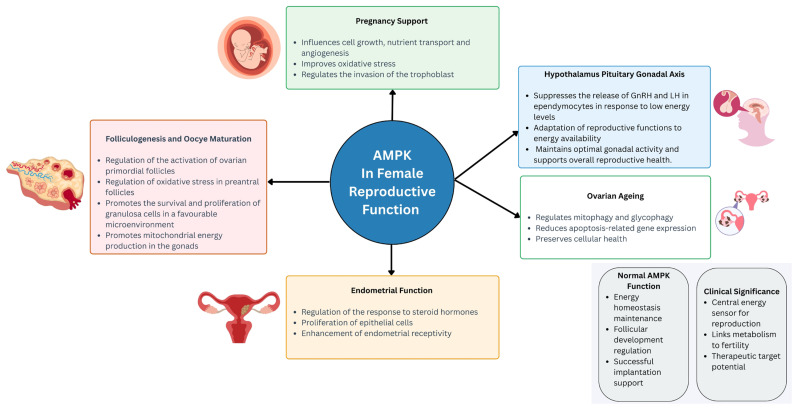
The physiological roles of AMPK in female reproductive function. Abbreviations: AMPK, AMP-activated protein kinase; GnRH, gonadotropin-releasing hormone; LH, luteinising hormone.

In another study, treatment with the plant flavonoid nobiletin (Nob) activated cell autophagy through AMPK and sirtuin-1 (SIRT1) in senescent SWF granulosa cells (SWF-GCs) induced by D-galactose. This activation restored the expression of proliferation-related molecules and proteins and reduced the inflammation-related protein NF-κB in senescent GCs. Nob could potentially prevent ovarian ageing by promoting mitophagy, antioxidant capacity, and reduced apoptosis-related gene expression and preserving cellular health through AMPK and SIRT1 activation in laying hens (Bai et al., 2024) [60]. Figure 1 summarises the role of AMPK in female reproductive function, while Table 1 summarises the studies addressing the role of AMPK in female reproductive function.

### 2.2. AMPK in Female Reproductive Diseases

#### 2.2.1. AMPK in Pre-Eclampsia

AMPK has received considerable attention as a potential therapeutic target for the prevention and treatment of pre-eclampsia due to its role in energy sensing and vascular homeostasis. Pre-eclampsia is a pregnancy-related condition characterised by a new onset of hypertension, typically associated with proteinuria and usually presenting after 20 weeks of pregnancy [61]. It affects around 4–5% of all pregnancies worldwide and is one of the most common complications of pregnancy [62,63,64,65].

In a rat model of pre-eclampsia, homeobox A9 (HOXA9) directly activates chemerin transcription, which subsequently triggers AMPK activation. This leads to the upregulation of thioredoxin-interacting protein (TXNIP) and activation of the NOD-like receptor family pyrin domain-containing 3 (NLRP3) inflammasome, promoting pyroptosis and inflammation in trophoblasts through the HOXA9-chemerin- chemokine-like receptor 1-AMPK-TXNIP-NLRP3 (HOXA9-chemerin-CMKLR1-AMPK-TXNIP-NLRP3) pathway [66]. This study showed that chemerin signalling via CMKLR1 activates AMPK and upregulates TXNIP expression in trophoblasts, which contributes to the inflammatory response. AMPK/TXNIP activation stimulates the NLRP3 inflammasome, increases interleukin(IL)-1β and IL-18 levels, and promotes trophoblast pyroptosis [67,68]. Importantly, the inhibition of chemerin or its downstream components (CMKLR1, HOXA9 and AMPK) reduced inflammation and pyroptosis, suggesting a promising therapeutic strategy for pre-eclampsia [66,69,70]. In support of these findings, clinical data showed that serum AMPK levels were significantly elevated in patients with severe PE compared to healthy pregnant women and those with non-severe PE, suggesting that AMPK not only mediates disease pathogenesis but may also serve as a biomarker for disease severity and prognosis [71]. This convergence of in vivo mechanistic data and clinical observations emphasises the translational importance of the chemerin–AMPK–TXNIP–NLRP3 axis in pre-eclampsia.

Similarly, Ma et al. (2021) investigated the role of AMPK in decidualised tissue from pre-eclampsia and healthy pregnancies [72]. Using an in vitro decidualisation model, they found that AMPK, ATP synthase (ATPS), and stress-70 protein (STRESS-70) regulate both cellular energy metabolism and the decidualisation process in pre-eclampsia [73]. Changes in the expression of cytidine triphosphate synthase (CTPS) during in vitro decidualisation were inversely related to AMPK signalling activity. The downregulation of CTPS led to impaired decidualisation and reduced AMPK signalling. During in vitro decidualisation, CTPS changes correlated with opposite fluctuations in AMPK signalling. On the third day of decidualisation, CTPS interacted with ATPS to maintain ATP levels, while its association with STRESS-70 decreased ATP production. Abnormal CTPS expression impairs decidualisation and has been linked to the pathogenesis of pre-eclampsia [72,74].

#### 2.2.2. AMPK in Premature Birth

A premature birth is defined as a live birth that occurs before 37 weeks of pregnancy [75]. Worldwide, around 15 million babies are affected, and the preterm birth rate is around 11% R. This condition is a major cause of mortality in children under the age of five, with around one million people dying each year as a result of premature birth. Low- and middle-income countries, particularly in Southeast Asia and sub-Saharan Africa, bear a disproportionate burden of these cases [76].

AMPK is involved in various pregnancy complications, including gestational diabetes mellitus, pre-eclampsia, intrauterine growth restriction, and premature birth [77]. The activation of the mTOR signalling pathway induces decidual cell senescence in early pregnancy, and phosphorylated mTOR increases cyclooxygenase-2 (COX2)-derived prostaglandin levels, leading to spontaneous preterm birth in 50–60% of p53-deficient mice [78,79]. In addition, AMPK activators such as AICAR, resveratrol, and metformin have been shown in preclinical studies to alleviate pregnancy complications. However, in mice with catechol-O-methyltransferase (COMT), an enzyme that metabolises catechol, metformin, failed to activate AMPK, suggesting that COMT is necessary for AMPK activation via 2-methoxyestradiol [80]. These findings suggest that AMPK may be a potential therapeutic target in pregnancy disorders, although excessive activation may pose a risk for foetal malformations [81].

A previous study has shown that the smooth muscle-specific deficiency of AMPK-α1/α2 leads to persistent pulmonary hypertension in neonates [82]. In addition, mice lacking both isoforms of AMPK-α1 or AMPK-α2 exhibited premature postnatal death, reduced alveolar number, thickened alveolar membranes without oedema, and extensive muscularisation and remodelling of the pulmonary artery tree. Interestingly, the deletion of AMPK-α1 or AMPK-α2 alone reduced hypoxic pulmonary vasoconstriction without inducing pulmonary hypertension, suggesting distinct roles for these isoforms in the regulation of pulmonary vasculature.

#### 2.2.3. AMPK in Polycystic Ovary Syndrome (PCOS)

Polycystic ovary syndrome (PCOS) is one of the most common hormonal disorders in women of reproductive age, affecting 5.5% to 19.9% of this population [83,84]. It is a combination of endocrine, metabolic, and reproductive disorders that lead to irregular menstrual cycles, dyslipidaemia, excessive body weight, oxidative stress, hyperandrogenism, and infertility [85,86]. In addition, around 40% of affected women are infertile, making this condition the most common cause of anovulatory infertility [87,88].

The regulatory effects of AMPK on metabolic and inflammatory processes were investigated in a letrozole-induced PCOS rat model [89]. It was shown that the pathophysiology of PCOS involves hormonal imbalance, metabolic dysfunction, and inflammation via the activation of nuclear factor kappa-light-chain-enhancer of activated B cells p65 subunit/IL-1β (NF-κB p65/IL-1β) and AMPK/PI3K/AKT, as well as the downregulation of nuclear factor erythroid 2–related factor 2 (Nrf2) and upregulation of the NLRP3 inflammasome [90,91]. In addition, histopathological analysis confirmed that treatment with fisetin, a polyphenolic flavonoid, alleviated PCOS symptoms, suggesting its therapeutic potential through the modulation of AMPK/PI3K/AKT-mediated antioxidant and inflammatory signalling [89].

Similarly, a recent study has demonstrated that Woxuanzhongzhou (WXZZ) alleviates insulin resistance and anovulation, restores the oestrus cycle, and reduces body weight and serum levels of testosterone, the luteinising hormone, and homeostatic model assessment for insulin resistance (HOMA-IR) [92]. These improvements were associated with increased irisin secretion via the upregulation of AMPK/peroxisome proliferator-activated receptor gamma coactivator-1 alpha/fibronectin type III domain-containing protein 5 (AMPK/PGC1-α/FNDC5) in skeletal muscle and CaMKK/AMPK/PGC1-α/uncoupling protein 1 (CaMKK/AMPK/PGC1-α/UCP1) in adipose tissue. These findings also show that the therapeutic effect of WXZZ in PCOS may be mediated by the activation of the AMPK/PGC1-α signalling pathway [93].

In a DHEA-induced PCOS rat model, AMPK is involved in autophagy and insulin resistance [94]. In primary granulosa cells, the AMPK/AKT/mTOR signalling pathway has been implicated in the regulation of autophagy and ovarian function. Treatment with berberine, metformin, or their combination affected autophagy-related proteins. In particular, granulosa cell autophagy was suppressed, as evidenced by the decreased expression of Beclin1 and LC3II/LC3I and increased expression of p62 [95]. These findings suggest that the activation of the AMPK/AKT/mTOR signalling pathway may play a key role in alleviating PCOS by modulating autophagy.

#### 2.2.4. AMPK in Endometriosis

Endometriosis is a widespread gynaecological disease that affects more than 10% of women and is one of the main causes of infertility and chronic pelvic pain. Historically, Rokitansky first described endometriosis in the 1860s as endometrial-like cells within the myometrium. In 1897, Cullen expanded this understanding by identifying severe lesions in the vaginal septum, which he termed adenomyomas, and Sampson (1921) further characterised the disease by describing “chocolate cysts” in the ovaries and classic black, furrowed lesions. Endometriotic lesions consist of clonal populations of specific cells that have different characteristics, such as progesterone resistance and aromatase activity [96].

In an in vivo mouse model of surgically induced endometriosis in female B6CBA/F1 mice, increased phospho-AMPKα expression was detected in the metformin-treated endometriosis (EM) group, indicating partial AMPK activation under oxidative conditions [97]. Although metformin increased GPx1 expression in endometriotic mice, other components of the AMPK/SIRT1/PGC-1α/SIRT3 signalling pathway in cardiac tissue remained largely unaffected. Thus, the antioxidant effect of metformin may be glutathione peroxidase 1 (GPx1)-specific and dependent on an antioxidant effect.

A recent study has shown that AMPK signalling is altered in endometriotic lesions in human endometrial tissue [98]. This dysregulation, particularly in perivascular and stromal cells, appears to promote metabolic reprogramming and lesion propagation. Similarly, Qi et al. (2022) proposed that the AMPK/mTOR signalling pathway mediates endometrial energy metabolism and receptivity in patients with a thin endometrium [99]. They observed that the downregulation of AMPK increases endometrial receptivity, promotes embryo implantation and improves pregnancy outcomes. Assaf et al. (2022) further emphasised that the disruption of AMPK/mTOR signalling promotes cell proliferation, migration, and lesion survival under energy stress and mitochondrial dysfunction [100]. Increased mitochondrial output and ROS production associated with hormonal and oxidative stress further drive disease progression [101]. These mechanisms provide a valuable framework for the development of future diagnostic and therapeutic approaches for endometriosis.

#### 2.2.5. AMPK in Dysmenorrhea

Dysmenorrhoea is defined as a common disorder in adolescent girls, young women, and those of school and university age [102,103]. It contributes significantly to absenteeism from school and work. Primary dysmenorrhoea is characterised by cramp-like pain in the lower abdomen and/or pelvis that occurs shortly before or during menstruation and is usually due to increased prostaglandin production without the presence of an underlying condition such as endometriosis [104]. In contrast, secondary dysmenorrhoea may be due to conditions such as endometriosis, structural pelvic abnormalities or infection and often manifests as progressively worsening pain, abnormal uterine bleeding, vaginal discharge, or dyspareunia [102].

**Figure 2:**
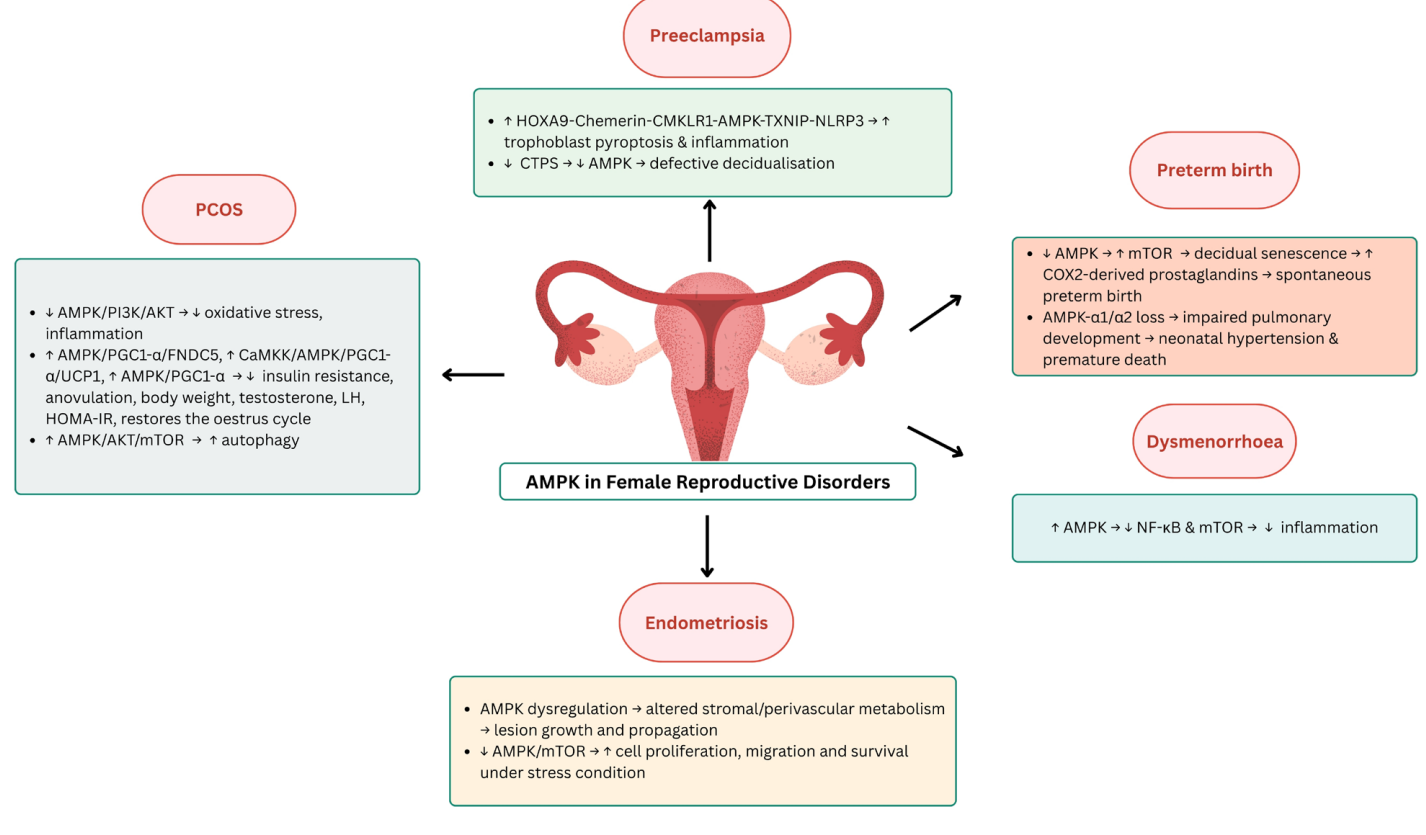
The role of AMPK in female reproductive disorders. Abbreviations: AKT, protein kinase B; AMPK, AMP-activated protein kinase; CaMKK, calcium/calmodulin-dependent protein kinase kinase; CMKLR1, chemerin chemokine-like receptor 1; CTPS, cytidine triphosphate synthase; COX2, cyclooxygenase-2; FNDC5, fibronectin type III domain-containing protein 5; HOMA-IR, homeostatic model assessment of insulin resistance; HOXA9, homeobox A9; LH, luteinizing hormone; mTOR, mechanistic target of rapamycin; NF-κB, nuclear factor kappa-light-chain-enhancer of activated B cells; NLRP3, NOD-like receptor family pyrin domain containing 3; PGC1-α, peroxisome proliferator-activated receptor gamma coactivator 1-alpha; PI3K, phosphoinositide 3-kinase; TXNIP, thioredoxin-interacting protein; UCP1, uncoupling protein 1.

AMPK activation by AICAR significantly inhibits IL-1β-induced inflammatory responses in primary cultured human endometrial stromal cells (ESCs) [105]. In particular, AICAR reduces the production of inflammatory cytokines (IL-8 and MCP-1) and prostaglandins (PGE2 and PGF2α) and inhibits the COX-2 expression and phosphorylation of downstream targets. This anti-inflammatory effect is attributed to the AMPK-mediated suppression of the NF-κB and mTOR signalling pathways. These results suggest that AMPK activators could be potential therapeutic candidates for the treatment of inflammatory gynaecological diseases such as dysmenorrhoea. Figure 2 summarises the role of AMPK in female reproductive diseases, while Table 2 summarises the studies addressing the role of AMPK in female reproductive diseases.

## 3. Limitations and Future Directions

To provide an appropriate context for interpreting the results and identifying areas for future research, several limitations of this review should be considered. First, although substantial mechanistic insights have been gained from in vivo and in vitro studies, there is a notable lack of clinical data directly validating these signalling pathways and targets in human populations. This gap limits the immediate translational relevance of the reviewed findings and emphasises the need for well-designed clinical studies to confirm these mechanistic relationships. In addition, species-specific differences need to be considered, particularly with respect to AMPK isoforms. Rodents and humans have different AMPK subunit composition and regulatory mechanisms, which may influence their responses to pharmacological agents and physiological stimuli. These interspecies differences may limit the extrapolation of the findings from animal models to human physiology and disease contexts.

To improve the understanding of AMPK signalling in reproductive health, several important research directions are of interest. One promising avenue is the investigation of epigenetic regulation by AMPK in reproductive cells. Future studies should investigate whether AMPK directly affects DNA methylation, histone modifications, and the expression of non-coding RNAs, especially under metabolic stress conditions, and how these epigenetic changes affect gametogenesis, implantation, and embryonic development. Another important area of research is the role of AMPK in mitochondrial function and energy metabolism in oocytes and spermatocytes. Investigating how AMPK modulates mitochondrial quality control, biogenesis, and oxidative stress may reveal therapeutic strategies to improve fertility and optimise the outcomes of assisted reproductive technologies (ARTs). In the context of age-related reproductive decline, targeted studies are needed to clarify how AMPK activity changes over time and whether the modulation of AMPK could delay or reverse reproductive decline in both sexes. It is important that clinical trials of AMPK modulators, both activators and inhibitors, are conducted to evaluate their therapeutic efficacy, safety, and specificity in the treatment of reproductive disorders, such as PCOS, endometriosis, and dysmenorrhea.

## 4. Conclusions

The AMP-activated protein kinase (AMPK) signalling pathway has been shown to be a central regulator of female reproductive physiology, closely linking cellular energy homeostasis to reproductive function. It plays a central role in critical reproductive processes such as folliculogenesis, oocyte maturation, the maintenance of pregnancy, and placental development. Its involvement in the reproductive system emphasises its importance in maintaining reproductive health. The dysregulation of AMPK signalling is increasingly recognised as a contributing factor to a spectrum of female reproductive disorders such as polycystic ovary syndrome (PCOS), endometriosis, infertility, and reproductive ageing, primarily through the disruption of metabolic and inflammatory balance. This review highlights the potential of targeting AMPK to restore physiological balance and treat these complex diseases. A better understanding of the multiple functions of AMPK could offer a promising pathway for novel interventions aimed at preventing and treating female reproductive dysfunction to ultimately improve fertility and enhance women’s reproductive wellbeing.

## Figures and Tables

**Table 1:** A summary of the studies addressing the role of AMPK in female reproductive function.

Experimental Model	Intervention	Key Findings	AMPK-Related Pathway	Reference
Rat model	2 µmol/8 µL, 200 µM AICAR infusion into 4th ventricle	↓ LH pulsatility ↑ ependymocytes Ca^2+^	AMPK	[16]
Ovariectomized, steroid-primed adult female Sprague–Dawley rats	12.5 U/kg subcutaneous insulin injection 25 µM/2.0 µL/h continuous infusion of L-lactate into the caudal fourth ventricle	↓ pAMPK, Fos protein profiles ↑ DbH protein	AMPK/GnRH	[19]
In vitro mouse ovaries	0, 3, 10, 30 and 100 μM compound C treatment for 0, 1, 4, 8, 24 and 96 h	↑ phosphorylation of ovarian mTOR, ribosomal protein S6, eIF4B ↓ TSC2 phosphorylation ↑ ovarian weights ↑ ovarian angiogenesis ↑ antral and preovulatory follicles ↑ Hif1a, Vegfa, Vegfr2 and Ctgf	AMPK/TSC2/mTOR/eIF4B/S6; AMPK/mTOR; AMPK/CTGF	[30]
In vitro juvenile mice ovaries	Dorsomorphin or metformin treatment	↑ activation of primordial follicles	AMPK/Foxo/Wnt	[34]
In vitro mice preantral follicles	2.5 nM astaxanthin treatment for 10 days	↑ antrum formation and maturation rates ↑ area of follicle attachment ↑ estradiol ↓ follicular malondialdehyde ↑ GSH, SOD ↓ ROS ↑ p-AMPK, PGC-1α, NRF2, HO-1, CO1, CO2, CO3, ATP6, ATP8, TOM20, PINK1, Parkin, LC3-II, Bcl-2, StAR, P450scc ↑ mitochondrial membrane potential ↓ caspase 3, Bax, P53	AMPK/PGC-1α, NRF2/HO-1; PINK1/Parkin/LC3-II; NRF1/TFAM; Bcl-2/Bax/P53/caspase 3; StAR/P450scc	[40,41]
In vitro maturation bovine oocyte	10, 20, 50, 100, and 200 μmol/L BAIBA treatment	↑ oocyte maturation ↑ CPT1A, CPT1B, CPT2 ↑ lipid metabolism ↓ lipid content ↑ mitochondrial membrane potential and active content	AMPK	[42]
Pregnant Kunming mice (F0 generation)	30 mg/kg/day polystyrene nanoplastics via intragastric administration from 0.5 gestation day to 21 days postpartum	↓ fertility of female F1 offspring ↑ rates of miscarriage and premature delivery ↓ litter size in the F0 generation ↓ primordial follicles ↑ growing follicles ↓ transzonal projections (TZPs) in the ovaries of adult F1 mice ↓ CAMKIIβ, Smad3 phosphorylation, E-cadherin ↑ oestrous phase duration ↓ diestrus phase duration ↓ serum levels of AMH and E2 in adult F1 progeny during proestrus ↓ body weight in offspring mice	AKT-FOXO3a	[43]
URSA mouse model (CBA/J × DBA/2)	50 mg/kg/day subcutaneous metformin for 14 days 2 mg and 8 mg/kg/day intraperitoneal 2-DG for 14 days	↓ rate of abortion ↓ cell degeneration and necrosis ↓ trophoblast inflammatory cell infiltration ↓ mTOR, GLUT1, and HK2 ↑ Foxp3 and IL-10, Tregs cells ↓ RORγt, IL-17, Th17 cells ↑ Treg/Th17 ratio	AMPK/mTOR	[53]
In vitro ageing chicken granulosa cells (GCs)	D-galactose (0, 12.5, 25, 50, 100, 200 mM) for 12 h or 24 h; FSH (0, 0.001, 0.01, 0.1, 1 IU/mL) for a further 24 h	Activates mitophagy Relieves mitochondrial oedema ↑ number of mitophagosomes ↑ mitochondrial light chain 3 (LC3)	AMPK-PI3K/AKT	[58]
Age-related diminished ovarian reserve mice	low dose (LD, 5.0 × 10^6^ cells/kg), middle dose (MD, 7.5 × 10^6^ cells/kg), and high dose (HD, 10.0 × 10^6^ cells/kg) of human amnion-derived mesenchymal stem cells (hAMSCs)	↑ ovarian function ↓ apoptosis of granulosa and stromal cells ↑ AMPK and the ratio of phosphorylated FoxO3a to total FoxO3a ↑ SOD2	AMPK/FoxO3a	[59]
In vitro D-galactose-generated senescent SWFs granulosa cells	1 to 100 μg/mL nobiletin treatment	Activates cell autophagy ↑ antioxidant capacity ↓ expression genes associated with cell apoptosis alleviates mitochondrial oedema	AMPK/SIRT1	[60]

Abbreviations: AICAR, 5-Aminoimidazole-4-carboxamide ribonucleotide, AKT, protein kinase B; AMPK, adenosine monophosphate (AMP)-activated protein kinase; ATP6/8, ATP synthase F0 subunit 6/8; Bax, Bcl-2-associated X protein; Bcl-2, B-cell lymphoma 2; CAMKIIβ, calcium/calmodulin-dependent protein kinase II beta; CPT, carnitine palmitoyltransferase;CTGF, connective tissue growth factor; DbH, dopamine β-hydroxylase; eIF4B, eukaryotic translation initiation factor 4B; E-cadherin, epithelial cadherin; FoxO3a, forkhead box O3a; Foxp3, forkhead box P3; GLUT1, glucose transporter 1; GnRH, gonadotropin-releasing hormone; HK2, hexokinase 2; Hif1a, hypoxia-inducible factor 1-alpha; HO-1, heme oxygenase 1; IL-, interleukin-; LC3-II, microtubule-associated protein 1 light chain 3-II; mTOR, mechanistic target of rapamycin; NF-κB, nuclear factor kappa-light-chain-enhancer of activated B cells; NRF1, nuclear respiratory factor 1; NRF2, nuclear factor erythroid 2-related factor 2; P53, tumor protein p53; Parkin, E3 ubiquitin-protein ligase Parkin; PGC-1α, peroxisome proliferator-activated receptor gamma coactivator 1-alpha; PINK1, PTEN-induced kinase 1; P450scc, cytochrome P450 side-chain cleavage enzyme; ROS, reactive oxygen species; RORγt, RAR-related orphan receptor gamma t; S6, ribosomal protein S6; SIRT1, sirtuin 1; Smad3, mothers against decapentaplegic homolog 3; SOD2, superoxide dismutase 2; StAR, steroidogenic acute regulatory protein; TOM20, translocase of outer mitochondrial membrane 20; Tregs, regulatory T cells; TSC2, tuberous sclerosis complex 2; TZPs, transzonal projections; UCP1, uncoupling protein 1; Vegfa, vascular endothelial growth factor A; Vegfr2, vascular endothelial growth factor receptor 2; ↑, increase; ↓, decrease.

**Table 2:** A summary of the studies addressing the role of AMPK in female reproductive diseases.

Experimental Model	Intervention	Key Findings	AMPK-Related Pathway	Reference
p-53 deficient mice model	1 mg/kg of oral metformin on days 8, 10, and 12 30 mg/kg of oral resveratrol on days 8, 10, 12, and 14	↓ premature decidual senescence ↓ spontaneous and inflammation-induced preterm birth ↓ AMPK and mTORC1 signalling in decidual cells	AMPK/mTOR	[78]
Letrozole-induced PCOS rat model	1.25 or 2.5 mg/kg/day of oral fisetin for 14 days	↓ LH and FSH ↑ AMH ↑ Nrf2 ↓ NLRP3	AMPK/PI3K/AKT; NLRP3/NF-κB p65/IL-1β	[89]
DHEA and HFD-induced PCOS-IR mice model	270 mg/kg/day of gavage WXZZ for 2 weeks	↓ body weight ↓ serum testosterone ↓ LH and LH/FSH ratio ↓ FINS ↓ HOMA-IR ↓ serum NEFA levels ↓ serum irisin levels ↓ lipid accumulation ↑ AMPK, PGC-1α, FNDC5, and irisin in gastrocnemius ↑ CaMKK, AMPK, PGC1-α, and UCP1 in subcutaneous fat	AMPK/PGC1-α/FDNC5; CaMKK/AMPK/UCP1	[92]
DHEA-induced PCOS rat model	150 mg/kg/day of gavage berberine, 300 mg/kg/day of gavage metformin or a combination of both for 30 days	↓ body weight ↓ ovarian weight ↑ number of primordial and primary follicles ↓ number of secondary and atretic follicles Normalised the oestrous cycle Improved insulin resistance, androgen biosynthesis, oxidative stress and lipid metabolism disorders ↑ oestrogen ↓ autophagosomes in granulosa cells ↓ Beclin1 and LC3II/LC3I levels ↑ p62	AMPK/AKT/mTOR	[94]
Surgically induced endometriosis female B6CBA/F1 mouse model	50 mg/kg/day of oral metformin for 3 months	↑ pAMPKα ↑ GPx1 ↑ miR-34a, miR-195, miR-155, and miR-421	AMPK/SIRT1/PGC1-α/SIRT3	[97]
Human endometrial stromal cells (ESCs)	AICAR treatment	↓ inflammatory cytokines (IL-8 and MCP-1) ↓ prostaglandins (PGE2 and PGF2α) ↓ COX-2 ↓ phosphorylation of IκB, 4EBP-1, p70S6K and S6 ribosomal protein	AMPK/NF-κB/mTOR	[105]

Abbreviations: 4EBP-1, eukaryotic translation initiation factor 4E-binding protein 1; AKT, protein kinase B; AMH, anti-Müllerian hormone; AMPK, adenosine monophosphate (AMP)-activated protein kinase; CaMKK, calcium/calmodulin-dependent protein kinase kinase; COX-2, cyclooxygenase-2; FINS, fasting insulin; FNDC5, fibronectin type III domain-containing protein 5; FSH, follicle-stimulating hormone; GPx1, glutathione peroxidase 1; HOMA-IR, homeostatic model assessment for insulin resistance; IκB, inhibitor of nuclear factor kappa B; IL-, interleukin-; LC3I/II, microtubule-associated protein 1 light chain 3-I/II; LH, luteinising hormone; MCP-1, monocyte chemoattractant protein-1; miR-, microRNA-; mTOR, mechanistic target of rapamycin; mTORC1, mechanistic target of rapamycin complex 1; NEFA, non-esterified fatty acids; NF-κB, nuclear factor kappa-light-chain-enhancer of activated B cells; NLRP3, NOD-like receptor family pyrin domain-containing 3; Nrf2, nuclear factor erythroid 2-related factor 2; P70S6K, ribosomal protein S6 kinase beta-1; PGE2, prostaglandin E2; PGF2α, prostaglandin F2 alpha; PGC1-α, peroxisome proliferator-activated receptor gamma coactivator 1-alpha; p62, sequestosome-1; phospho-AMPKα, phosphorylated AMP-activated protein kinase alpha subunit; S6, ribosomal protein S6; SIRT, sirtuin; UCP1, uncoupling protein 1; ↑, increase; ↓, decrease.
